# An Improved Memetic Algorithm for Detecting Protein Complexes in Protein Interaction Networks

**DOI:** 10.3389/fgene.2021.794354

**Published:** 2021-12-14

**Authors:** Rongquan Wang, Huimin Ma, Caixia Wang

**Affiliations:** ^1^ School of Computer and Communication Engineering, University of Science and Technology Beijing, Beijing, China; ^2^ School of International Economics, China Foreign Affairs University, Beijing, China

**Keywords:** protein complexes, protein-protein interaction networks, memetic algorithm, fitness function, graph clustering methods

## Abstract

Identifying the protein complexes in protein-protein interaction (PPI) networks is essential for understanding cellular organization and biological processes. To address the high false positive/negative rates of PPI networks and detect protein complexes with multiple topological structures, we developed a novel improved memetic algorithm (IMA). IMA first combines the topological and biological properties to obtain a weighted PPI network with reduced noise. Next, it integrates various clustering results to construct the initial populations. Furthermore, a fitness function is designed based on the five topological properties of the protein complexes. Finally, we describe the rest of our IMA method, which primarily consists of four steps: selection operator, recombination operator, local optimization strategy, and updating the population operator. In particular, IMA is a combination of genetic algorithm and a local optimization strategy, which has a strong global search ability, and searches for local optimal solutions effectively. The experimental results demonstrate that IMA performs much better than the base methods and existing state-of-the-art techniques. The source code and datasets of the IMA can be found at https://github.com/RongquanWang/IMA.

## 1 Introduction

Many complex systems in the real world are often modeled with complex networks, such as computer networks, social networks, and biological networks. The detection of community structure is an essential property of complex networks, and it helps us understand the structure and functionally of complex networks. Protein complexes mined from the PPI network are representative of detecting community structure in complex networks. In proteomics, proteins rarely act alone, and often organize together to form protein complexes to perform specific biological functions cooperatively (Spirin and Mirny, 2003). Therefore, accurately identifying protein complexes from PPI networks can contribute to the study of the mechanisms of cellular functions and organization (Gavin et al., 2002) in the post-genomic era. Although some experimental methods such as yeast two-hybrid and tandem affinity purification can detect protein complexes, they have limitations. Specifically, they are expensive and time-consuming. With the development of high-throughput experimental technologies, many PPI networks are now available. The computational methods developed complement the experimental techniques in identifying protein complexes. As a result, many computational methods have been proposed for the identification of protein complexes from PPI networks, which is a type of cluster analysis, which consists of grouping patterns into clusters based on the similarity, and it is a valuable technology in many areas such as bioinformatics, machine learning, and computer vision.

To date, a variety of computational methods for detecting protein complexes in PPI networks have been proposed. Based on our work, we provide a summary of the related works by classifying the existing methods into three types: methods based on reducing noise, methods based on different topological structures, and methods based on evolutionary algorithms.

Because the PPI networks are derived from high-throughput experiments, the high false positive and false negative rates in the PPI networks are high (Von Mering et al., 2002; Samanta and Liang, 2003; Liu et al., 2009; Srihari, 2012; Zaki et al., 2013; Lei et al., 2018), which substantially affects the accuracy of protein complex identification. To reduce the influence of noise, some methods utilize various network topological properties for strong interactions and identify protein complexes. These algorithms include PEWCC (Zaki et al., 2013), ProRank+ (Hanna and Zaki, 2014), and EWCA (Wang et al., 2019a). Alternatively, some studies have attempted to integrate the gene expression data (Keretsu and Sarmah, 2016), gene ontology data (Zhang et al., 2013a; Wang et al., 2019b), and subcellular localization data (Wang et al., 2020) to improve the reliability of the interactions. These studies mainly use biological data to weight protein interactions and compensate the PPI network, which better reflects the real protein interactions. DPCT (SabziNezhad and Jalili, 2020) uses TAP and GO data to construct a weighted PPI network and to reduce the noise of PPI, and a memetic algorithm to detect protein complexes. Valdeolivas et al. (2019) extended the Random walk with restart (RWR) algorithm to multiplex and heterogeneous networks. This framework performs better as compared to the aggregation of the different interaction sources and the multiplex framework is more efficient than network aggregations to extract communities from biological networks. Blatti et al. (2020) presented Knowledge Engine for Genomics (KnowEnG), it is a free-to-use computational system for analysis of genomics data sets, it provides the standard clustering pipeline to cluster a collection of samples. However, further reducing the impact of random noise in PPI networks, and improving the performance of protein complex detection methods remain urgent problems to be solved.

In recent years, various computational methods based on different topological structures have been developed to detect protein complexes in PPI networks. Among these methods, different types of topological structures are commonly assumed to be protein complexes. Some methods partition proteins into many non-overlapping clusters by using partition functions or principles. For example, Markov Clustering (MCL) (Van Dongen, 2000) is a simulated random walk method, which mainly uses expansion and inflation operators to manipulate the adjacency matrix and mine protein complexes from the PPI networks. Meanwhile, RNSC (King et al., 2004) first moves the proteins randomly among the clusters to optimize the cost function, and then a post process, based on cluster size, density, and functional homogeneity is carried out. Some methods aim to find cliques. In 2006, CFinder (King et al., 2004) was developed to cluster proteins in the PPI network, and it used the concept of k-clique to discover protein complexes. In 2009, CMC (Liu et al., 2009) tried to enumerate cliques in the PPI network for discovering protein complexes, but it was too strict for most protein complexes. Therefore, several methods based on density have been designed to identify dense subgraphs in the PPI networks, where subgraphs with densities above a pre-defined threshold were considered as protein complexes. For example, MCODE (Bader and Hogue, 2003) first weighted every node by local neighborhood density and then extended locally dense nodes to detect protein complexes. Later, Li et al. (2008) improved the seed-extended method by modifying the DPClus algorithm based on the diameter and density of the local graph. Furthermore, Gavin et al. (2006) proposed that protein complexes have the core-attachment structure, and some identification methods based on the core-attachment structure. For example, COACH (Wu et al., 2009) and WPNCA (Peng et al., 2014) have been proposed to find protein complexes. They first extracted the protein complex cores from the neighborhood graphs of the proteins, and protein complex cores were further extended to form complete protein complexes. Finally, some protein complexes with a high overlap were merged. There are some variants of network topological features that are used to detect protein complexes; these studies (Nepusz et al., 2012; Giurgiu et al., 2019) have shown that proteins in a protein complex commonly display strong interactions within the core of the protein complex, and weak interactions with the proteins outer surface of the protein complex, ClusterONE (Nepusz et al., 2012) starts from a seed node and inserts neighbors into it to form overlapping protein complexes by using cohesiveness. Subsequently, Wang et al. (2019b) proposed a novel seed-expand algorithm called SE-DMTG to identify protein complexes with a combinatorial function from the weighted PPI networks. Additionally, based on the 3-sigma principle (Wang et al., 2013), MPC-C (Wang et al., 2020) identifies the active points of proteins in a time serial of gene expression data and generates a series of time-sequenced subnetworks to identify static and dynamic protein complexes. In 2021, Liu et al. (2021) proposed a protein complex detection methods based on a semi-supervised model to detect protein complexes with clear module structures. A number of computational methods only consider single topological properties to identify protein complexes, and they recover protein complexes with other types of topological structures.

Intensive studies on evolutionary algorithms have also been conducted. In recent years, some researchers have provided new ideas for solving protein complex identification problems by using optimized algorithms, by employing the characteristics of highly adaptive and good optimization abilities. Some successful methods have been applied to tackle the problems of identifying protein complexes and efficiency. In 2015, Ramadan et al. (2016) introduced a genetic algorithm to detect protein complexes. Subsequently, in 2016, Lei et al. (2016) presented F-MCL based on Markov clustering and the firefly method, which automatically determines the parameters by using the firefly method. In the same year, a novel fruit fly optimization clustering method was designed to detect dynamic protein complexes (Lei et al., 2016). In 2017, Zhang et al. (2017) proposed a new firefly clustering algorithm for transforming the protein complex detection problem into an optimization problem. Zhao et al. (2017) proposed a novel improved cuckoo search clustering method for discovering protein complexes in dynamic weighted PPI networks. In 2019, Lei et al. (2019b) used a nature-inspired optimization method to detect protein complexes. In 2019, a moth-flame-optimization-based protein complex detection method was presented (Lei et al., 2019a). In 2020, an evolutionary algorithm based on a heuristic biological operator was introduced to detect protein complexes (Abduljabbar et al., 2020). These evolutionary methods have a strong global search ability, but they have difficulty in locating the local optima efficiently.

To solve these issues, we present a novel algorithm, named IMA, which uses an improved memetic algorithm we designed to detect protein complexes from the PPI network. First, we constructed a weighted PPI network by using the common neighbor, gene expression data, GO-slim data, and subcellular location data to reduce the impact of noise on our IMA. Second, many high-quality initial individuals, including protein complexes with different topological structures, are generated using EWCA (Wang et al., 2019a), SE-DMTG (Wang et al., 2019b), and MPC-C (Wang et al., 2020). We propose a fitness function to identify protein complexes with various topological properties. Third, a new improved memetic algorithm is proposed to mine the protein complexes by optimizing this fitness function in the weighted PPI network. Remarkably, its selection, recombination, and updating population operators are used for the global search of the best individual, and a local optimization strategy is designed to locate the local optima individually. Finally, our IMA was applied to four different yeast PPI networks and compared with the 12 existing excellent methods. The experimental results illustrate that the IMA achieves state-of-the-art performance of computational metrics and biological relevance metrics in identifying protein complexes.

In the materials and methods section, we introduce the datasets and standard protein complexes used in the evaluation of IMA and define all phases of IMA separately. In the experiments and results section, we evaluated the proposed method and compare it with the state-of-the-art methods. The case study and discussion section shows some examples of protein complexes detected by IMA and we conclude this paper in conclusion section.

## 2 Materials and Methods

### 2.1 Datasets

In this study, we used four PPI networks including Krogan (Krogan et al., 2006), DIP (Xenarios et al., 2002), combined6 (Liu et al., 2009), and WI-PHI (Kiemer et al., 2007). Their information is presented in Table 1.

**TABLE 1:** Statistics of used four PPI networks in the study.

Dataset	Number of node	Number of edge	Density
Krogan	2674	7075	0.0019796849348
DIP	4930	17201	0.0014157219124
combined6	3869	17327	0.0023156247135
WI-PHI	5955	49604	0.0027980540426

Furthermore, two sets of standard protein complexes from the literature (Wang et al., 2020) were used to evaluate the performance of the protein complex detection methods, and their information is shown in Table 2. Here, standard protein complexes 1 consists of the known protein complexes from MIPS (Mewes et al., 2004), SGD (Hong et al., 2007), TAP06 (Gavin et al., 2006), ALOY (Aloy et al., 2004), CYC 2008 (Pu et al., 2009) and NEWMIPS (Friedel et al., 2009). Meanwhile, standard protein complexes 2 also is a combined protein complexes dataset (Ma et al., 2017). It consists of the Wodak database (Pu et al., 2009), PINdb and GO complexes (Ma et al., 2017). Additionally, we also used CYC2008 protein complexes (Pu et al., 2009) and MIPS protein complexes (Mewes et al., 2004), and they come from other people’s work, and they are shown in https://github.com/RongquanWang/IMA/Additional file 3.

**TABLE 2:** Statistics of used standard protein complexes.

Datasets	Number	Protein coverage	Avg size
Standard protein complexes 1	812	2773	8.92
Standard protein complexes 2	1045	2778	8.97
CYC2008 protein complexes	193	1371	8.33
MIPS protein complexes	212	1202	15.61

The GO-slim data can explain the biological function of proteins, and it can be downloaded from https://downloads.yeastgenome.org/curation/literature/go_slim_mapping.tab. The gene expression data were obtained from https://www.ncbi.nlm.nih.gov/sites/GDSbrowser. Additionally, the subcellular localization data set for yeast proteins was obtained from https://compartments.jensenlab.org/Downloads.

### 2.2 Methods

#### 2.2.1 Preliminaries

Since PPI networks are defined using graph-theoretic concepts, we first provide some of the terminologies used in our paper and then describe the IMA method in detail.

A PPI network can be represented as an undirected graph *G*(*V*, *E*), where *V* is the set of vertices (individual proteins) and *E* is the set of edges (protein interactions) between the vertices. The neighbors of *v* in *G*, denoted by *N*(*v*), are the set of vertices adjacent to *v*.

Biologically, protein complexes are groups of proteins that interact with each other at the same time and place, forming a single multi-molecular machine. However, due to the inherent topological structures of protein complexes (Nepusz et al., 2012) in PPI networks, protein complexes are usually assumed to be the subgraphs of PPI networks. Let *C* = (*V*_*C*_, *E*_*C*_, *W*_*C*_) be a subgraph of *G*. The neighbors of *C* are defined by Eq.1:

$$N\left(C\right)=\left\{v|\left(v,u\right)\in E,u\in V_{C},v\in V-V_{C}\right\},$$ (1)

As a result, the task of identifying protein complexes can be formulated as mining connected clusters that are densely connected inside and well separated from the rest of the PPI networks, and the clusters correspond to the protein complexes. The protein complex detection method obtains a set of clusters, *P* = (*P*_1_, *P*_2_, …, *P*_*t*_).

#### 2.2.2 Methods

##### 2.2.2.1 Constructing a Weighted PPI Network

Many studies (Lei et al., 2018, 2019b; Wang et al., 2020) have shown that the performance of protein complex identification methods can be improved by integrating multiple data sources into a single weighted PPI network, which enhances the confidence of interactions in PPI networks. Thus, we integrated the topological structures and multiple biological data to weight the interactions in the PPI networks. Our goal was to weight the edges of the PPI network to reflect the reliability of the corresponding interactions. Graph clustering algorithms can use these weights to reduce the influence of noisy edges and yield meaningful clusters.

###### 2.2.2.1.1 Protein Common Neighbor similarity

The larger the number of common neighbors between the two proteins, the stronger the interaction of the two proteins, and they are more likely to participate in the same protein complex. Some common neighbor similarity measures (Zaki et al., 2013; Wang et al., 2019a; Wang et al., 2019b) were used to calculate the similarity of protein pairs. This paper defines a higher-order common neighbor (HCN) similarity measure to estimate the reliability of the interaction between two proteins, *v* and *u*. The HCN is defined by Eq. 2.

$$HCN\left(v,u\right)=\sqrt{\frac{|NCN\left(v,u\right){|}^{2}}{\sqrt{\left|N\left(v\right)|*|N\left(u\right)\right|}*|N\left(v\right)\cup N\left(v\right)|}},$$ (2)

where *NCN*(*v*, *u*) = *N*(*v*) ∩ *N*(*u*) is the number of common neighbors between proteins *v* and *u*. *N*(*v*) and *N*(*u*) represent the number of neighbors that proteins *v* and *u* are connected with, respectively. *HCN*(*v*, *u*) can further balance the comprehensive connectivity of the two interacting proteins, which may consist of the same protein complex.

###### 2.2.2.1.2 Protein Co-Expression similarity

Next, the gene expression data describes proteins under various conditions in a biological process (Zhang et al., 2013b; Wang et al., 2014). The gene expression vector of each protein comprised of a series of expression values within the period. If two proteins have a similar degree of expression at the same time interval, they have a high co-expression value, and then they are more likely to form a protein complex. The gene expression profiles of a pair of proteins *v* and *u* in a PPI network, their gene expression profiles are *v* = {*v*_1_, *v*_2_, …, *v*_*n*_} and *u* = {*u*_1_, *u*_2_, …, *u*_*n*_}, respectively. Here, we use the person correlation coefficient (Wang et al., 2013) to calculate the co-expression value *PCC*(*v*, *u*), as defined in Eq. 3:

$$PCC\left(v,u\right)=\frac{\sum_{i=1}^{n}\left(v_{i}-\bar{v}\right)*\left(u_{i}-\bar{u}\right)}{\sqrt{\sum_{i=1}^{n}{\left(v_{i}-\bar{v}\right)}^{2}}*\sqrt{\sum_{i=1}^{n}{\left(u_{i}-\bar{u}\right)}^{2}}},$$ (3)

where $\bar{v}$ and $\bar{u}$ are the average gene expressions of proteins *v* and *u* in *n* time points, respectively. *PCC*(*v*, *u*) indicates the co-expression of the vector representation between two interacting proteins. As the value of *PCC*(*v*, *u*) ranges from −1 to 1, we set $PCC\left(v,u\right)=\frac{PCC\left(v,u\right)+1.0}{2}$ to set *PCC*(*v*, *u*) in [0,1]. The higher the value of *PCC*(*v*, *u*), the larger the probability of co-expression of proteins *v* and *u* and formation of a protein complex.

###### 2.2.2.1.3 Protein Functional Similarity

From the perspective of protein function, we used GO-slim data to reflect the functional similarity of proteins. Moreover, we generated an attribute matrix *O* ∈ *R* ^*N*×*M*^, where *N* denotes the number of proteins in the PPI network and *M* denotes the number of GO slim attributes. Based on matrix *O*, we constructed a protein attribute affinity matrix, *S* ∈ *R* ^*N*×*N*^. Each entry *FS*(*v*, *u*) reflects the GO slim attribute similarity between proteins *v* and *u*, as defined in Eq. 4:

$$FS\left(v,u\right)=\frac{\sum_{k=1}^{M}o_{vk}*o_{uk}}{\sqrt{\sum_{k=1}^{M}o_{vk}^{2}}*\sqrt{\sum_{k=1}^{M}o_{uk}^{2}}}.$$ (4)

###### 2.2.2.1.4 Protein Subcellular Location Similarity

Generally, if two interacting proteins have the same subcellular location, the interaction between the proteins is more reliable. Proteins in the protein complex should be localized in the same inner cellular compartment. Here, we defined the subcellular location similarity *SL*(*v*, *u*), and is defined in Eq. 5:

$$SL\left(v,u\right)=\frac{|SL\left(v\right)\cap SL\left(u\right){|}^{2}}{\left|SL\left(v\right)|*|SL\left(u\right)\right|}$$ (5)

where |*SL*(*v*)| and |*SL*(*u*)| denote the number of subcellular localizations of proteins *v* and *u*, respectively. |*SL*(*v*) ∩ *SL*(*u*)| represents the number of common subcellular localization attributes between proteins *v* and *u*.

The edges whose weight is 0 are seen as noise and are removed from the PPI network, and the rest of edges whose weight *W*(*v*, *u*) are expressed by Eq. 6:

$$W\left(v,u\right)=\frac{HCN\left(v,u\right)+PCC\left(v,u\right)+FS\left(v,u\right)+SL\left(v,u\right)}{4}.$$ (6)

Finally, the weighted PPI networks were constructed, and the reliability of the PPI networks was enhanced.

##### 2.2.2.2 Memetic Algorithm

A memetic algorithm (Li et al., 2014) is typically a hybrid-local heuristic search method used for optimization. Generally, memetic algorithms consist of a genetic algorithm and local optimization strategy. Here, the genetic algorithm is the global search method, which can explore a reliable estimate of the global optimum, but it does not obtain an optimal individual in the explored search space quickly. Therefore, the local optimal search strategy is typically used to accelerate searching and find the best individual in the local search space. In this paper, we present an improved memetic algorithm that can create new individuals that are located in new promising regions in the global search space and search around the newly generated individuals to optimize individuals of better quality (Gach and Hao, 2012).

##### 2.2.2.3 The IMA Algorithm for Complex Detection

###### 2.2.2.3.1 The Framework of IMA Algorithm

**FIGURE 1:**
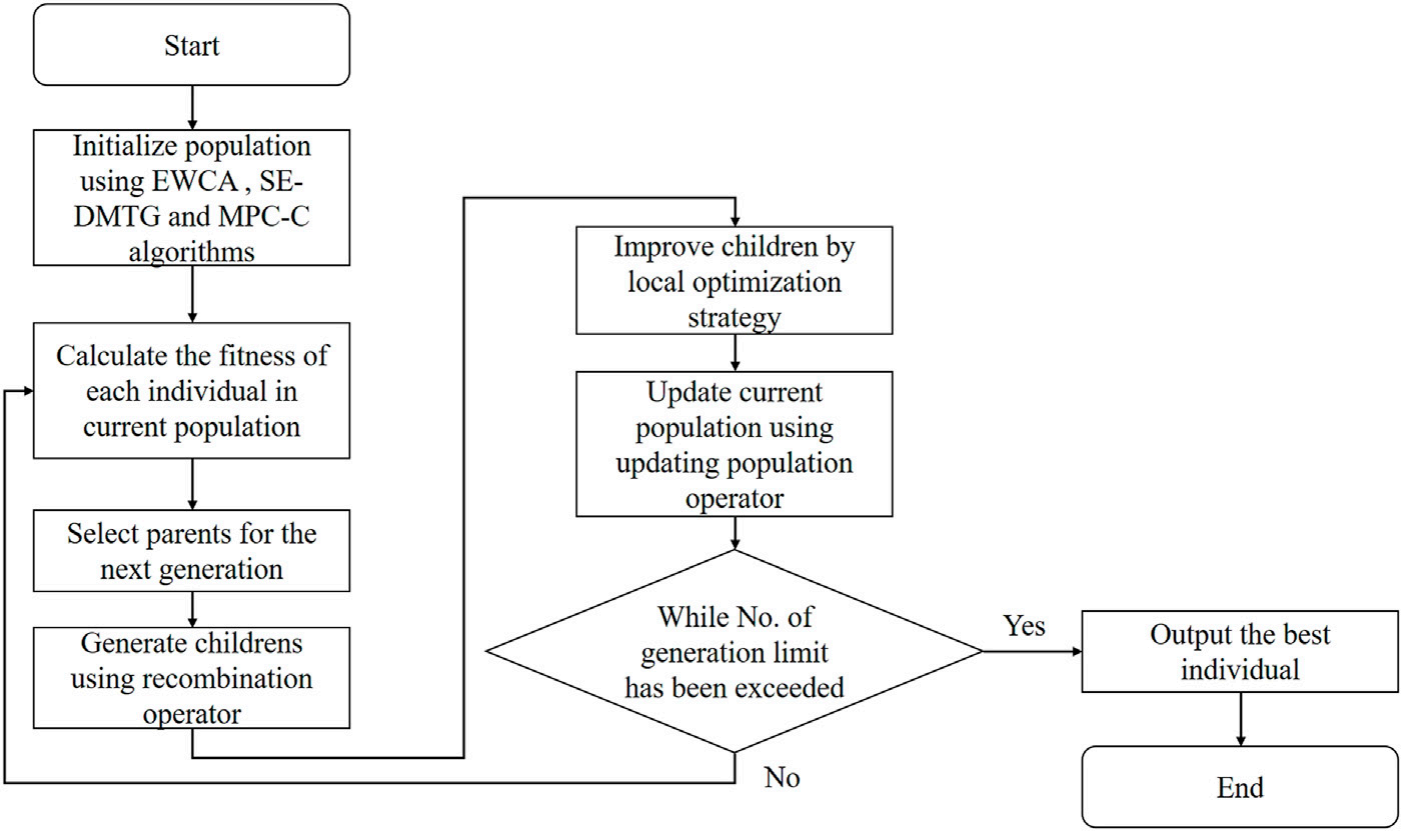
The flowchart of IMA framework.

The memetic algorithm is a valuable framework for dealing with combinatorial optimization problems. It provides a good balance between searching for diversification and optimization by employing a genetic algorithm and the local optimization strategy (Žalik and Žalik, 2018). We designed a fitness function to evaluate the quality of the clusters. In addition, we propose a recombination operator and local optimization strategy for detecting protein complexes in the PPI network. Our goal is to maximize the individual’s *FS*_*fitness*_ (see Eq. 13) using an improved memetic algorithm. Algorithm 1 is the main framework of IMA. The primary input of the IMA algorithm was the PPI network. A flowchart of Algorithm 1 is presented in Figure 1.

IMA begins with initial populations (line 1, see section generating the initial population) and then repeats an iterative procedure many times (generations) (lines 3–18). Two parent individuals in the current population are selected using a tournament selection strategy for each generation (line 4, see section selection operator). Two new children individuals were generated based on two selected parents using the recombination operator (line 5, see section recombination operator). Then, we select the children with the highest fitness function (line 6, see section fitness function: Eq. 13), and if its fitness function is larger than the 80*%* fitness of the individual with the maximum fitness, it is further improved using the local optimization strategy we proposed (line 7–11, see section local optimization strategy). Finally, a roulette wheel selection strategy is used to update the population (line 12, see section updating population operator). The individual with the highest fitness function (line 13, Eq. 13) mined during the search process is always recorded (lines 14–16). The entire IMA stops when *iter* consecutive generations (line 3, *Max*_*Iter*_). In the following subsections, we provide more details on the parts of the IMA algorithm.

###### 2.2.2.3.2 Generating Initial Population

As we know, most of existing protein complex detection methods are based on unsupervised learning, and they can only identify protein complexes with a single topology. However, real protein complexes have a variety of topologies. That is why we proposed the IMA. In fact, IMA algorithm is a typical swarm intelligence optimization algorithm, which needs to build an initial population *P*. In order to build an initial population, we should follow two basic requirements: 1) The individuals in the initial population should be high quality. It means that the protein complex detection method should produce the high accuracy initial population; 2) Individuals in the initial population need to be diverse. Obtaining initial population method should identify the protein complexes with different topological structures. Based on the above two points, we choose EWCA (Wang et al., 2019a), SE-DMTG (Wang et al., 2019b) and MPC-C (Wang et al., 2020) as the methods to generate the initial population.

In fact, according to their references, we can see that these three algorithms not only have high protein complex detection accuracy, but also can identify the protein complexes with different topological structures. And their advantageous than other existing methods are shown in their literatures (Wang et al., 2019a; b, 2020). For example, EWCA can identify protein complexes with core-attachment structure, SE-DMTG can identify protein complexes with high density and modularity and MPC-C can predict static protein complexes and dynamic protein complexes with community structure. Moreover, the identification accuracy of these three algorithms is also excellent in the existing algorithms. Therefore, they can ensure the generation of protein complexes with high quality and different topological structures. That is why we chose these algorithms.

The first approach is the EWCA, which generates *o* individuals by using different values of *ss* (structural similarity) threshold and ranges from 0.3 to 0.62 with 0.04 increment. The second approach is the SE-DMTG algorithm, which generates *o* individuals by randomly constructing seed queues, and then uses its seed-extended algorithm to generate individuals. The last method is the MPC-C algorithm, which is used to construct *o* individuals by setting different values of the filter *Score*(*C*) cutoff (from 0.1 to 0.8 with a step size of 0.1). Finally, we obtain *Pop* = 3 × *o* individuals and combine them to create the initial population, where the parameter *o* is 8.

Algorithm 1 The pseudo-code of general framework of IMA.

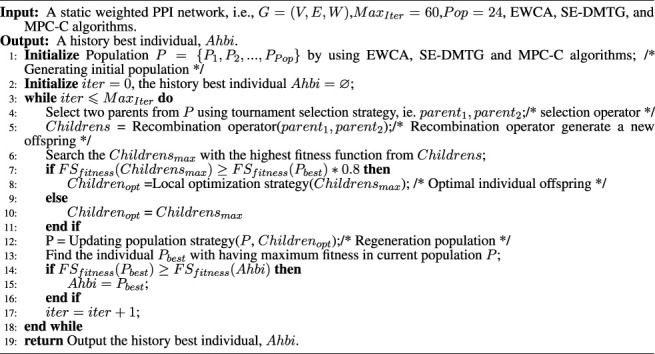

###### 2.2.2.3.3 Fitness Function

We first define the fitness function of the IMA method; this fitness function should reflect the topological properties of protein complexes in PPI networks. Generally, a high-quality protein complex is a group of proteins that are densely interconnected but only sparsely connected with the rest of the PPI network (Li et al., 2008; Nepusz et al., 2012; Wang et al., 2019b, 2020). Meanwhile, in identifying various topological properties of protein complexes, the combination of multiple single objective functions can compensate for the shortcomings of a single objective function, which leads to an improved quality of the identified protein complexes. Therefore, we propose a multi-objective function (Eq. 12) by integrating five objective functions (Eqs 7–11 to describe the topological properties of protein complexes: *cohesiveness*(*C*), *density*(*C*), *AIEW*(*C*), *ABEW*(*C*), and *AWM*(*C*).

*C* = (*V*_*C*_, *E*_*C*_, *W*_*C*_), where *V*_*C*_ is the set of proteins in cluster *C*, *E*_*C*_ is the set of interactions in cluster *C*, and *W*_*C*_ is the set of weights between the pair of proteins. According to previous studies (Nepusz et al., 2012; Wang et al., 2019b), the cohesiveness score is defined as Eq. 7:

$$cohesiveness\left(C\right)=\frac{W_{in}\left(C\right)}{W_{in}\left(C\right)+W_{out}\left(C\right)},$$ (7)

where *W*_*in*_(*C*) is the sum of the weights of all edges among *C*, and *W*_*out*_(*C*) is the sum of the weights of the edges connecting nodes in *C* to other nodes in the rest of the PPI network.

(Li et al., 2008; Liu et al., 2009; Wang et al., 2019b) hypothesized that the higher the density of the cluster is, the more likely the cluster is a protein complex in the PPI network. The weighted density of cluster *C* is defined by Eq. 8:

$$density\left(C\right)=\frac{2*W_{in}\left(C\right)}{|V_{C}|*\left(|V_{C}|-1\right)}$$ (8)

where *W*_*in*_(*C*) is the sum of the weights of the edges between them, and *V*_*C*_ is the number of nodes in *C*.

In this paper, we propose three measures to estimate the likelihood of a local cluster *C* being a protein complex. First, it is the average inner edge weight (AIEW), and it can estimate the reliability of the internal edges of the cluster *C*. This is defined in Eq. 9:

$$AIEW\left(C\right)=\frac{W_{in}\left(C\right)}{\left|E_{C}\right|},$$ (9)

where *W*_*in*_(*C*) is the sum of the weights of the edges among them, and |*E*_*C*_| is the number of edges in cluster *C*. *AIEW*(*C*) is the average weight of the inner edges in cluster *C*.

Second, it is the average border edge weight (ABEW), and it can measure the reliability of the border edges of cluster *C*. This is defined in Eq. 10:

$$ABEW\left(C\right)=\frac{W_{out}\left(C\right)}{\left|BE_{C}\right|},$$ (10)

where |*BE*_*C*_| = {(*u*, *v*)|*u* ∈ *C*, *v*∉*C*} is the number of border edges that connect the cluster *C* with the rest of the PPI network, and *W*_*out*_(*C*) is the sum of the weights of the edges connecting nodes in *C* to the neighbor of the cluster *C*. *ABEW*(*C*) is the average weight of the border edges in cluster *C*.

Third, it is the average weighted modularity (AWM), which indicates that the cluster *C* is highly average weight connected among them and has a low average weight interaction with the rest of the network. This is defined in Eq. 11:

$$AWM\left(C\right)=\frac{AIEW\left(C\right)}{AIEW\left(C\right)+ABEW\left(C\right)},$$ (11)

Based on these objective functions, we propose a fitness function that combines these single objective functions to assess the possibility of a cluster *C* being a protein complex. This fitness function is denoted by Eq. 12:

$$FF\left(C\right)=cohesiveness\left(C\right)+density\left(C\right)+AIEW\left(C\right)-ABEW\left(C\right)+AWM\left(C\right),$$ (12)

here, the *density*(*C*) and *AIEW*(*C*) seek a dense intra-connection topological structure, whereas *ABEW*(*C*) identifies the sparse topological structure inter-connecting with the rest of the PPI network. *cohesiveness*(*C*) and *AWM*(*C*) are used to identify the topological structures with densely interconnected nodes that are sparsely connected to the rest of the PPI network.

As a result, *FF*(*C*_*i*_) is used to identify protein complexes with various topological structures in the individual *FS*. Finally, the fitness function (*FS*_*fitness*_) of the individual is the sum of *FF*(*C*_*i*_), which is defined in Eq. 13:

$$FS_{\mathrm{fi}tness}=\overset{k}{\underset{i=1}{\sum }}FF\left(C_{i}\right),$$ (13)

where *C*_*i*_ represents the *i*th cluster in the individual *FS*. *FF*(*C*_*i*_) is a multi-objective function that is designed to capture the community structure of protein complexes (a group of nodes has better internal connectivity than external connectivity). *k* is the total number of protein complexes found in an individual *FS*.

In this study, the goal of our IMA method is to find the individual with the maximum value of *FS*_*fitness*_. Generally, the higher the *FS*_*fitness*_ of an individual *FS*, and the better the quality of the individual. Therefore, the protein complex detection problem can be regarded as an optimization problem by maximizing the value of *FS*_*fitness*_ in a PPI network.

###### 2.2.2.3.4 Selection Operator

The selection operator is an essential operation used in the memetic algorithm. The main idea of the selection strategy is that the better an individual, the higher is its chance of being a parent. In the IMA algorithm, the recombination operator and updating population operator need individual selection strategies.

In the parent selection operator, a selection strategy is required to select two parent individuals from the current population to generate individual children. The fundamental principle of this operation is that individuals with a higher fitness function are more likely to be selected as parent individuals. Here, we use a binary tournament selection strategy to select the parent individuals. Meanwhile, the recombination operator also uses a binary tournament selection strategy to select protein complexes from composite parent individuals to create individual children.

Lastly, the population updating strategy needs to update the population according to the fitness of individuals in current population and generate children individuals. In this updating process, we use the roulette wheel selection strategy to update the current population to balance the relatively good individuals and avoid precocity.

###### 2.2.2.3.5 Recombination Operator

The recombination operator is the critical diversification mechanism of memetic algorithm. An effective recombination operator should generate not only diversified solutions but also transfer significant components from parents to children (Hao, 2012; Li et al., 2014). The recombination operator is responsible for combining the genetic material of several individuals (usually two individuals) to create a new children (Spears and De Jong, 1995). New children inherit many high-quality protein complexes from their parents. Additionally, the recombination operator plays a vital role in the effectiveness of the memetic algorithm in the global search space. Traditional recombination operators, having uniform crossover and two-point crossover, are challenging to convey the excellent protein complexes of two parents to a new children simultaneously. This method is less suitable for protein complex detection. Therefore, we present a recombination operator based on the fitness function, whose children can inherit the better protein complexes of their parents. This operator plays an essential role in preventing the algorithm from being trapped in an optimal local solution and exploring the global search space. The main idea of this operator is to take the protein complexes from two parents as the genetic material and try to retain the high-quality protein complexes in parents for the new children. The recombination operator is described in Algorithm 2.

Algorithm 2 Recombination operator.

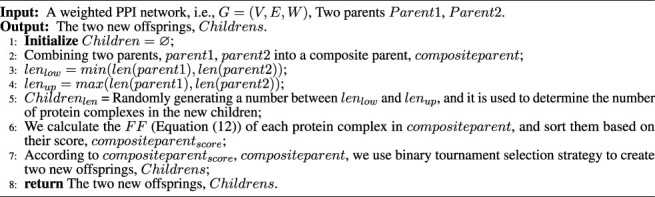

Let *parent*1 and *parent*2 represent the parents, and let *len*(*parent*1) and *len*(*parent*2) be the number of protein complexes in each parent. Parents *parent*1 and *parent*2 are merged into a composite parent. Note that if there are redundant protein complexes, and we only leave one. As a result, we obtain a composite parent individual *compositeparent* in line 2. Based on the length of *parent*1 (*len*(*parent*1)) and the length of *parent*2 (*len*(*parent*2)), we determine the length of the new children (*children*_*len*_) by randomly generating a number between *len*_*low*_ and *len*_*up*_ in lines 3–5. The multi-objective function (Eq. 12) of each protein complex in the *compositeparent* individual is calculated and sorted in line 6. Next, according to their multi-objective function (Eq. 12) and *compositeparent*, we use the binary tournament selection strategy to create two new offspring in line 7.

###### 2.2.2.3.6 Local Optimization strategy

To improve the quality of the generated offspring, we presented a new local optimization strategy to obtain better offspring. This strategy is different from the general sense of local search strategies, such as the hill-climbing strategy and simulated annealing strategy. The purpose of the local optimization strategy is to obtain offspring of relatively high quality. Here, each protein complex in children is optimized using a multi-objective function (Eq. 12) and a local optimization strategy. The local optimization strategy of the IMA is applied to new children as shown in Algorithm 3.

In this process, for each protein complex *Childrens*_*i*_ in the *Childrens*_max_, we optimize it using the following steps in lines 3–22. First, we find inner nodes (*Innernodes*) that belong to the *Childrens*_*i*_ and connect at least one protein in the rest of the PPI networks in line 9. Then, we find the *inner*_*max* by improving the multi-objective function (see Eq. 12) maximum in *Innernodes* in line 10, and then we remove the *inner*_*max* from *Clusterdel* in line 11. Second, we find boundary nodes (*Boundarynodes*), which is the set of proteins that connect at least one inner protein of the current *Childrens*_*i*_ in line 15. Then, we detect the *boundary*_max_ by increasing the multi-objective function (see Eq. 12) maximum in line 16, and we insert the *boundary*_max_ into *Clusteradd* in line 17. We repeat the above two steps until the protein complex *Childrens*_*i*_ does no change, and if it is not changed (it is considered a locally optimal cluster) or *iteration* > 20 in lines 19–22, it is an identified protein complex *Opcs*_*i*_ in line 24. Next, we use the local optimization strategy to optimize the rest of the protein complexes in *Childrens*_max_ in line 2–25. If the fitness function of the optimized individual *Opcs* is not larger than that of the *Childrens*_max_, for the individual *Childrens*_max_, its local optimization strategy is deemed invalid in lines 26–29. Finally, we output this optimal child, *Opcs*, in line 30.

Algorithm 3 The local optimization strategy.

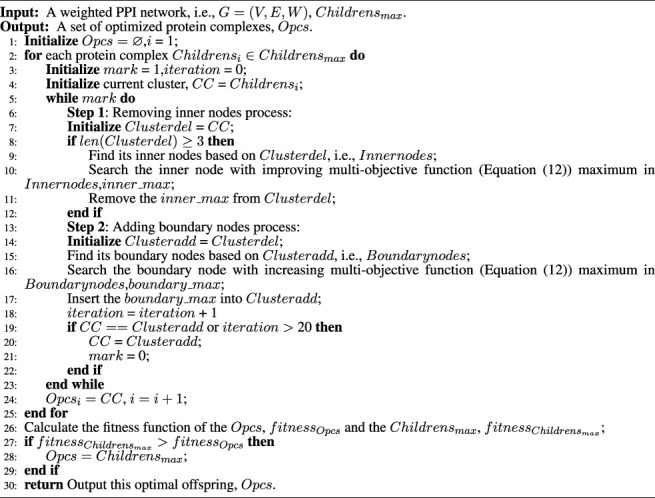

###### 2.2.2.3.7 Updating Population Operator

Population diversity is also a vital issue for memetic algorithms to effectively avoid prematurity. When a new child individual is produced with the recombination operator and local optimization strategy, the fitness function (Eq. 13) of the new children and the current population are calculated, respectively. All of these are sorted by their fitness functions. Moreover, to avoid premature convergence, we employ a roulette wheel selection strategy to update the population. Here, the size of the new population in each iteration is the same as that of the original population. The roulette wheel selection strategy can balance the diversity of the population and guarantee that individuals with higher fitness always have a greater probability of being retained in the population.

## 3 Experiments and Results

In the experiment, our operational environment was a windows 10 operating system with an Intel(R) Core(TM) i7-9700 CPU with a physical memory of 16 GB, and a speed of the processor was 3.60 GHz. The IMA was run on PyCharm Community Edition 2017.2.2. The IMA was implemented using the Python 3.

### 3.1 Evaluation Metrics

There are several statistical matching-based metrics that estimate the quality of the detected protein complexes based on different protein complex detection methods. Meanwhile, biological relevance-based metrics, which are supplementary to statistical matching-based metrics, are used to evaluate the biological significance of identified protein complexes.

If a detected protein complex *ipc* and a known protein complex *kpc* contain common proteins each other, their overlapping score (*OS*(*ipc*, *kpc*)) is calculated using Eq. 14:

$$OS\left(ipc,kpc\right)=\frac{|V_{ipc}\cap V_{kpc}{|}^{2}}{\left|V_{ipc}|\times |V_{kpc}\right|},$$ (14)

where *ipc* and *kpc* are the protein set of *ipc* and the protein set of *kpc*, respectively. If *OS*(*ipc*, *kpc*) ≥ *λ*, *ipc* is matched with *kpc*, where *λ* is a threshold.

The F-measure is the harmonic mean of precision and recall, and it can be calculated using Eq. 15:

$$F-measure=\frac{2\times precision\times recall}{precision+recall},$$ (15)

For more details, please see (Lei et al., 2019a). An identified protein complex is considered to match a standard protein complex where the overlap score *OS*(*ipc*, *kpc*) is larger than 0.2 (Lei et al., 2019a).

The coverage rate (CR) was used to measure the number of proteins in the standard protein complexes that could be covered by the identified protein complexes (Peng et al., 2014). This is defined in Eq. 16:

$$CR=\frac{\sum_{s=1}^{\left|S\right|}\max\left\{T_{st}\right\}}{\sum_{s=1}^{\left|S\right|}N_{s}},$$ (16)

For more details on these parameters, please refer to reference (Peng et al., 2014).

Generally, a higher *Sn* indicates that the identified protein complexes cover the proteins in the standard protein complexes better. In contrast, a higher *PPV* indicates that the identified protein complexes are more likely to be actual protein complexes. Accuracy (ACC) is the geometric average of *PPV* and *Sn*, which is denoted by Eq. 17:

$$\begin{array}{c} ACC=\sqrt{Sn\times PPV}. \end{array}$$ (17)

The maximum matching ratio (MMR) (Nepusz et al., 2012) can measure the overlap matching between standard protein complexes and detected protein complexes based on maximal one-to-one mapping. It can deal with the case that a known protein complex split into different parts in the identified protein complexes, because only one part is matched with the known protein complex.

*Jaccard* (Wang et al., 2019b) was used to quantify the overlap between the detected protein complexes and known protein complexes. In fact, *Jaccard* is defined as the harmonic mean of the *JaccardC* of the identified protein complexes and the *JaccardG* of standard protein complexes, and it is used to evaluate the clustering results. *Jaccard* is calculated using Eq. 18:

$$Jaccard=\frac{2\times \left(JaccardC\times JaccardG\right)}{JaccardC+JaccardG}.$$ (18)

As a result, the performance of the detection method is evaluated by the total score, which is calculated using Eq. 19 (Wang et al., 2020):

Totalscore=F−measure+CR+ACC+MMR+Jaccard. (19)

In this paper, the *p*-value is used to estimate the biological relevance of the identified protein complexes, and it is denoted by Eq. 20:

p−value=1−∑k=0K−1FkN−FC−kNC, (20)

For a more detailed explanation of these parameters, please refer to references (Lei et al., 2019a; Wang et al., 2020). If the *p*-value of the protein complex is less than 0.01, it means that the protein complex has biological significance.

The co-localization score (*CL*) (Krumsiek et al., 2008) is denoted as the maximal fraction of proteins in a protein complex that is found at the same location (Friedel et al., 2009). For all the detected protein complexes by different methods, the average co-localization score was computed using Eq. 21:

$$CL=\frac{\sum_{j=1}^{m}max_{i=1}^{n}l_{i,j}}{\sum_{j=1}^{m}N_{j}},$$ (21)

where *l*_*i*,*j*_ is the number of proteins in the detected protein complex *j* allocated to the localization group *i*, *N*_*j*_ is the number of proteins in the detected protein complex *j*, and *m* and *n* are the number of detected protein complexes and localization groups, respectively. The final localization score was calculated as the geometric mean of the co-localization scores based on the Huh datasets (Huh et al., 2003).

### 3.2 The Effects of Parameters

**FIGURE 2:**
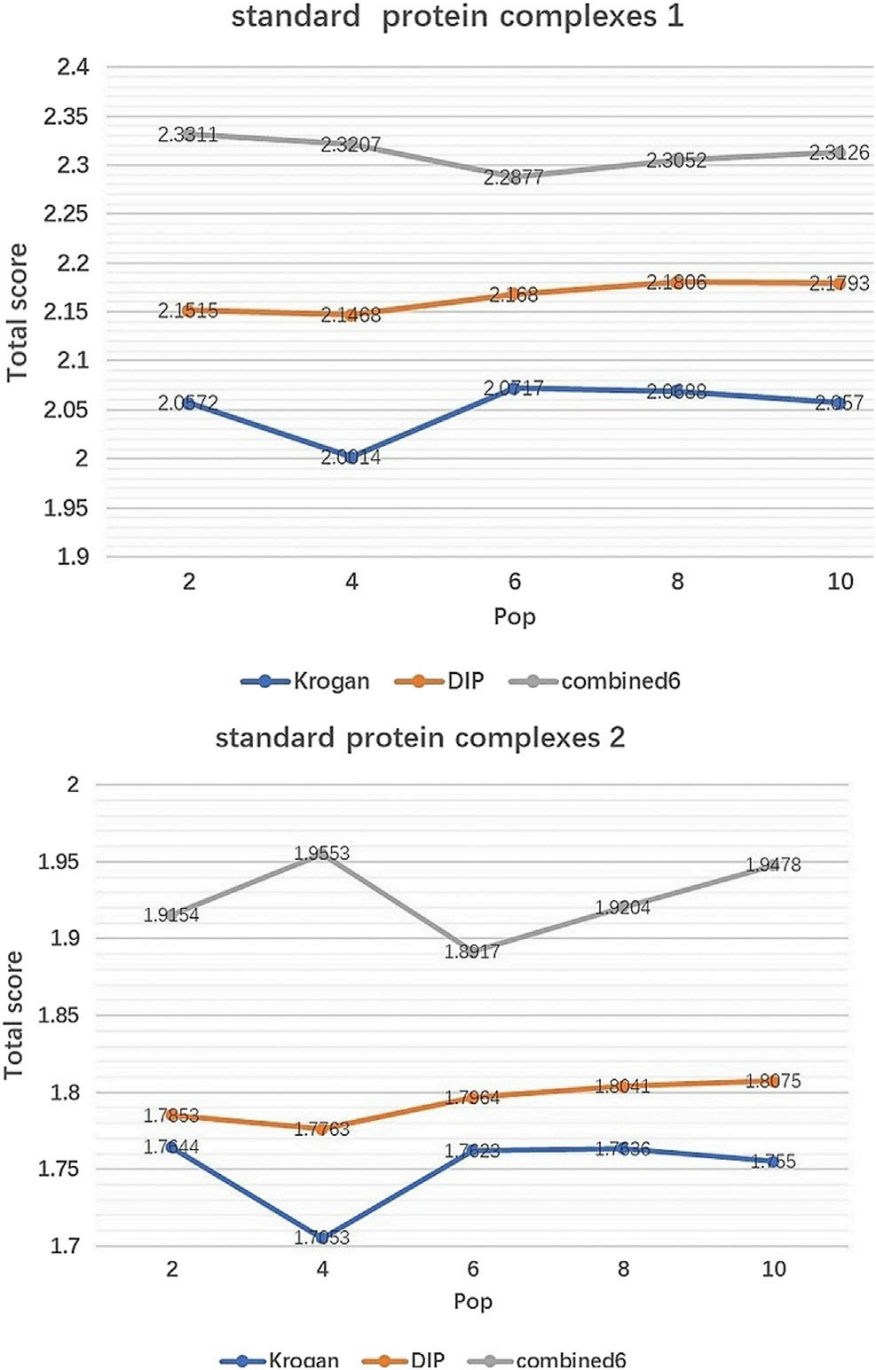
The effect of Pop. It shows how the variation of parameter Pop affect the Total score of IMA method in standard protein complexes 1 and standard protein complexes 2.

**FIGURE 3:**
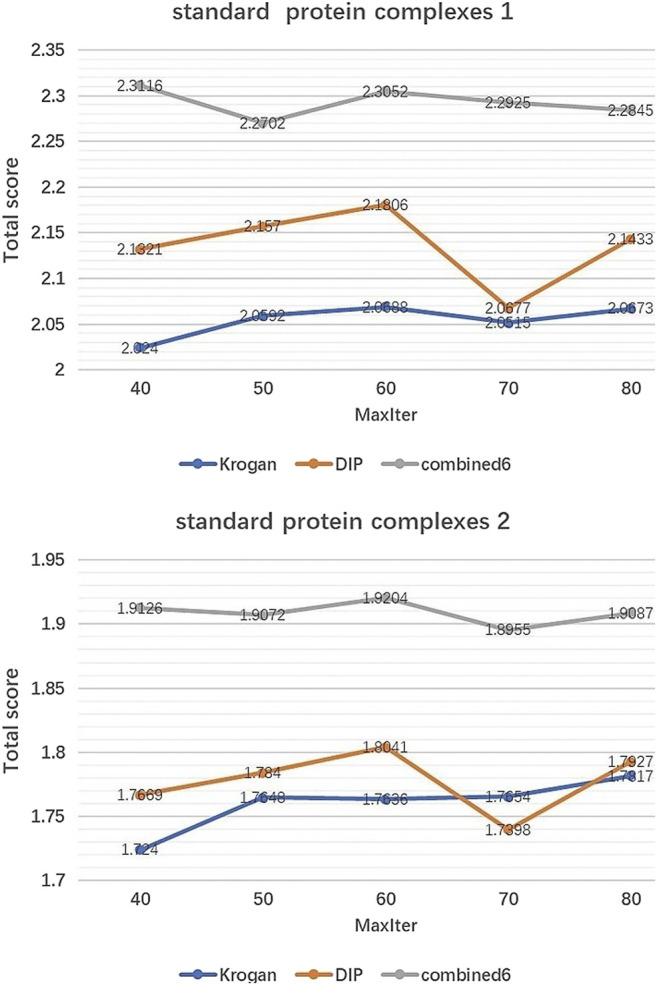
The effect of MaxIter. It shows how the variation of parameter MaxIter affect the Total score of IMA method in standard protein complexes 1 and standard protein complexes 2.

IMA includes two parameters which need to be tuned:Generations(MaxIter) and Population size (Pop). Pop controls the number of initial population by EWCA, SE-DMTG, MPC-C. MaxIter determines the number of iterations of population optimization. Figures 2, 3 show how performance is influenced by these parameters in three PPI networks and two standard protein complexes.

Effect of Pop. Figure 2 shows how Total score changes with the value of Pop. We can see that Total score is not very sensitive to Pop, especially when Pop falls in [6, 10]. So in our experiments, Pop is set to 8 by default.

Effect of MaxIter. Figure 3 shows the changing trend of Total score when MaxIter increases from 40 to 80. We can see that for the three PINs, the fluctuations of Total score are not significant. In our experiments, we set MaxIter = 60 by default.

According to Figures 2, 3, we can see that the Total score is not very sensitive to the changing trend of MaxIter and Pop, so we only set MaxIter = 60 and Pop = 8 as the default value. To avoid evaluation bias and overestimation of the performance, we do not tune the parameter to a particular dataset and set them as the default value in the all experiments. For more details on parameters setting, please see https://github.com/RongquanWang/IMA/Additional file 1.rar.

#### 3.2.1 Comparison With Competitive Algorithms

To demonstrate the performance of the IMA, we compared it with 12 state-of-the-art methods. These include MCODE (Bader and Hogue, 2003), MCL (Van Dongen, 2000), IPCA (Li et al., 2008), COACH (Wu et al., 2009), ClusterONE (Nepusz et al., 2012), PEWCC (Zaki et al., 2013), ProRank+ (Hanna and Zaki, 2014), WPNCA (Peng et al., 2014), WEC (Keretsu and Sarmah, 2016), EWCA (Wang et al., 2019a), SE-DMTG (Wang et al., 2019b), and MPC-C (Wang et al., 2020). The total score is used for a more comprehensive evaluation of the different methods. We obtained the software implementations for all the compared methods, and their parameters are shown in Table 3. Although better results could probably be obtained by fine-tuning these parameters, we only use default or suggestion thresholds to maintain the fairness of different algorithms.

**TABLE 3:** Parameters of each method used in the study.

ID	Algorithms	Parameter
1	MCODE	(default setting)
2	MCL	inflation = 2 (author suggestions)
3	IPCA	S = 3, *p* = 2,*T*_*in*_ = 0.6 (author suggestions)
4	COACH	w = 0.225 (default setting)
5	ClusterONE	Density = auto, Overlap threshold = 0.8 (author suggestions)
6	PEWCC	Overlap = 0.8,-r = 0.1,Re-join = 0.3 (author suggestions)
7	ProRank+	AdjstCD threshold = 0.45 (author suggestions)
8	WPNCA	lambda = 0.3,size = 3 (author suggestions)
9	WEC	Balance factor (*λ*) = 0.8, Edge weight (*T*_*w*_) = 0.7, Enrichment (*T*_*e*_) = 0.8, Filtering (*T*_*f*_) = 0.9 (author suggestions)
10	EWCA	Structural similarity (ss = 0.4) (author suggestions)
11	SE-DMTG	Size = 3 (author suggestions)
12	MPC-C	Overlap threshold = 0.8 (author suggestions)
13	IMA	Generations (*Max*_*Iter*_) = 60, Population size (*Pop*) = 24 (default setting)

The performance of the methods was compared on four PPI networks based on two standard protein complexes. The experimental results of our IMA and other methods on these PPI networks are listed in Tables 4, 5, and the highest value of each metric of each PPI network is in bold.

**TABLE 4:** Performance of different algorithms with respect to standard protein complexes 1.

Algorithms	Num	F-measure	CR	ACC	MMR	Jaccard	Total score
Krogan							
MCODE	39	0.3414	0.2140	0.1994	0.0351	0.2359	1.0258
MCL	370	0.4004	0.3895	**0.3192**	0.1123	0.2902	1.5117
IPCA	582	0.5573	0.3389	0.2653	0.2368	0.3713	1.7696
COACH	345	0.5254	0.3473	0.2667	0.1824	0.3556	1.6775
ClusterONE	240	0.4694	0.3085	0.2829	0.1262	0.3324	1.5194
PEWCC	383	0.5289	0.3231	0.2554	0.2194	0.3786	1.7053
ProRank+	357	0.5448	0.3660	0.2718	0.2018	0.3544	1.7388
WPNCA	369	0.5446	0.3897	0.2758	0.1663	0.3415	1.7179
WEC	516	0.5440	0.3442	0.2637	0.2573	0.4005	1.8089
EWCA	676	0.5883	0.3782	0.2769	0.3019	0.4073	1.9525
SE-DMTG	372	0.5878	0.3504	0.2820	0.2284	0.4058	1.8781
MPC-C	458	0.6010	0.3760	0.2814	0.2344	0.3882	1.8810
IMA-unweighted	767	0.5917	0.3827	0.2843	0.3221	0.4174	1.9982
IMA	773	**0.6272**	**0.3917**	0.2859	**0.3266**	**0.4373**	**2.0688**
DIP							
MCODE	26	0.1300	0.2193	0.1337	0.0103	0.1292	0.6224
MCL	628	0.3106	0.3578	0.2684	0.0752	0.2155	1.2275
IPCA	1242	0.5741	0.3519	0.2404	0.2096	0.3004	1.6764
COACH	329	0.5850	0.3697	0.2462	0.1254	0.3305	1.6568
ClusterONE	904	0.5118	**0.5062**	**0.3270**	0.1467	0.3297	1.8214
PEWCC	648	0.6004	0.3783	0.2438	0.1938	0.3514	1.7677
ProRank+	167	0.3123	0.2115	0.1870	0.0452	0.2007	0.9567
WPNCA	623	0.5888	0.4307	0.2594	0.1807	0.3360	1.7955
WEC	253	0.4185	0.3104	0.2309	0.0953	0.3078	1.3628
EWCA	964	0.6428	0.4374	0.2691	0.2534	0.3723	1.9750
SE-DMTG	869	0.6309	0.3822	0.2674	0.2264	0.3573	1.8482
MPC-C	1477	0.6632	0.4413	0.2729	0.2716	0.3537	2.0027
IMA-unweighted	1569	0.6861	0.4488	0.2731	0.2957	0.3959	2.0995
IMA	1338	**0.7196**	0.4528	0.2820	**0.3028**	**0.4234**	**2.1806**
combined6							
MCODE	63	0.2483	0.3441	0.1762	0.0313	0.1832	0.9833
MCL	508	0.3606	0.4628	0.3098	0.0909	0.2871	1.5114
IPCA	2160	0.7218	**0.5106**	0.2783	0.3093	0.4396	2.2597
COACH	682	0.5623	0.4839	0.2653	0.2035	0.3703	1.8855
ClusterONE	648	0.4165	0.5098	**0.3306**	0.1235	0.3173	1.6979
PEWCC	737	0.6586	0.4713	0.2594	0.2661	0.4370	2.0924
ProRank+	472	0.5837	0.3898	0.2394	0.2162	0.4363	1.8657
WPNCA	898	0.5912	0.5725	0.2720	0.1872	0.3306	1.9537
WEC	544	0.5614	0.4367	0.2504	0.2001	0.4120	1.8609
EWCA	935	0.6860	0.5058	0.2771	0.3097	0.4534	2.2321
SE-DMTG	490	0.6854	0.4347	0.2767	0.2326	0.4517	2.2321
MPC-C	1008	0.7001	0.4871	0.2769	0.2820	0.4438	2.1899
IMA-unweighted	1183	0.7097	0.4746	0.2771	0.3341	0.4755	2.271
IMA	1054	**0.7256**	0.4829	0.2734	**0.3364**	**0.4869**	**2.3052**
WI-PHI							
MCODE	124	0.1095	0.4282	0.1720	0.0142	0.1095	0.8333
MCL	772	0.2597	0.4323	0.2960	0.0647	0.2246	1.2773
IPCA	2181	0.5361	0.5789	0.2819	0.2604	0.3585	2.0156
COACH	1353	0.4689	0.6095	0.2752	0.1803	0.3144	1.8483
ClusterONE	1313	0.1813	0.4908	0.3103	0.0689	0.2065	1.2577
PEWCC	1813	0.5440	0.5943	0.2757	0.2535	0.3516	2.0192
ProRank+	255	0.1814	0.2038	0.1801	0.0259	0.1719	0.7630
WPNCA	1813	0.5385	0.6198	0.2834	0.2257	0.3432	2.0106
WEC	729	0.3700	0.4830	0.2353	0.0969	0.2987	1.4839
EWCA	964	0.6428	0.4374	0.2691	0.2534	0.3723	1.9750
SE-DMTG	774	0.4945	0.5198	**0.3107**	0.1816	0.3827	1.8894
MPC-C	2560	0.6068	0.5054	0.2793	0.2013	0.3668	1.9597
IMA-unweighted	3316	0.6769	0.5924	0.2983	0.3841	0.4423	2.3941
IMA	2561	**0.7503**	**0.6223**	0.3060	**0.3965**	**0.4828**	**2.5579**

The bold values are the highest value of each metric of each PPI network.

**TABLE 5:** Performance of different algorithms with respect to standard protein complexes 2.

Algorithms	Num	F-measure	CR	ACC	MMR	Jaccard	Total score
Krogan							
MCODE	39	0.2317	0.1863	0.1861	0.0238	0.1982	0.8260
MCL	370	0.3214	0.3534	**0.3088**	0.0792	0.2559	1.3187
IPCA	582	0.4606	0.3097	0.2405	0.1545	0.3271	1.4924
COACH	345	0.4369	0.3166	0.2441	0.1228	0.3136	1.4340
ClusterONE	240	0.3913	0.2729	0.2756	0.0887	0.2826	1.3110
PEWCC	383	0.4228	0.2913	0.2343	0.1418	0.3247	1.4150
ProRank+	357	0.4435	0.3282	0.2621	0.1306	0.3067	1.4711
WPNCA	369	0.4361	**0.3572**	0.2614	0.1083	0.2960	1.4590
WEC	516	0.4344	0.3022	0.2465	0.1559	0.3351	1.4741
EWCA	676	0.5112	0.3483	0.2663	0.1986	0.3607	1.6851
SE-DMTG	372	0.5060	0.3092	0.2684	0.1445	0.3471	1.5852
MPC-C	458	0.5252	0.3354	0.2706	0.1583	0.3338	1.6233
IMA-unweighted	767	0.5091	0.3488	0.2709	0.2078	0.3665	1.7031
IMA	773	**0.5452**	0.3526	0.2733	**0.2135**	**0.3790**	**1.7636**
DIP							
MCODE	26	0.1061	0.1982	0.1205	0.0071	0.1114	0.5433
MCL	628	0.2409	0.3025	0.2504	0.0482	0.1921	1.0341
IPCA	1242	0.4516	0.3196	0.2304	0.1298	0.2674	1.3989
COACH	329	0.4703	0.3184	0.2307	0.0800	0.2829	1.3823
ClusterONE	904	0.4232	**0.4358**	0.2937	0.0972	0.2874	1.5373
PEWCC	648	0.4812	0.3336	0.2329	0.1125	0.2986	1.4588
ProRank+	167	0.2506	0.1895	0.1802	0.0323	0.1784	0.8310
WPNCA	623	0.4603	0.3709	0.2472	0.1065	0.2866	1.4715
WEC	253	0.2921	0.2588	0.2422	0.0526	0.2497	1.0954
EWCA	964	0.5334	0.3812	0.2536	0.1522	0.3226	1.6429
SE-DMTG	869	0.5305	0.3403	0.2562	0.1382	0.3108	1.5697
MPC-C	1477	0.5692	0.3799	0.2538	0.1706	0.3050	1.6785
IMA-unweighted	1569	0.5763	0.3873	0.2583	0.1855	0.336	1.7435
IMA	1338	**0.6064**	0.3828	**0.2710**	**0.1894**	**0.3545**	**1.8041**
combined6							
MCODE	63	0.1771	0.2943	0.1642	0.0213	0.1543	0.8113
MCL	508	0.2902	0.4078	0.2966	0.0629	0.2605	1.3181
IPCA	2160	0.5641	0.4521	0.2824	0.1854	0.3725	1.8567
COACH	682	0.4454	0.4171	0.2626	0.1229	0.3184	1.5665
ClusterONE	648	0.3454	0.4385	**0.3145**	0.0885	0.2881	1.4752
PEWCC	737	0.5223	0.4064	0.2588	0.1531	0.3739	1.7145
ProRank+	472	0.4697	0.3305	0.2322	0.1237	0.3631	1.5194
WPNCA	898	0.4968	**0.5117**	0.2822	0.1182	0.3140	1.7231
WEC	544	0.4324	0.3842	0.2577	0.1172	0.3562	1.5478
EWCA	935	0.5657	0.4523	0.2810	0.1846	0.3971	1.8807
SE-DMTG	490	0.5568	0.3747	0.2782	0.1414	0.3793	1.7306
MPC-C	1008	0.5964	0.4077	0.2677	0.1752	0.3756	1.8225
IMA-unweighted	1183	0.602	0.3999	0.2695	0.1993	0.3988	1.8694
IMA	1054	**0.6127**	0.4138	0.2828	**0.2046**	**0.4066**	**1.9204**
WI-PHI							
MCODE	124	0.0766	0.3701	0.1606	0.0086	0.0938	0.7096
MCL	772	0.2116	0.3563	0.2776	0.0445	0.2042	1.0941
IPCA	2181	0.4655	0.4970	0.2830	0.1634	0.3250	1.7340
COACH	1353	0.3577	0.5228	0.2559	0.1115	0.2821	1.5300
ClusterONE	1313	0.1571	0.4179	0.2920	0.0481	0.1956	1.1108
PEWCC	1813	0.4464	0.5215	0.2615	0.1495	0.3189	1.6979
ProRank+	255	0.1397	0.1732	0.1714	0.0190	0.1502	0.6536
WPNCA	1813	0.4285	**0.5445**	0.2776	0.1346	0.3093	1.6945
WEC	729	0.2914	0.4329	0.2523	0.0590	0.2767	1.3122
EWCA	2347	0.4346	0.5382	0.2840	0.1679	0.3295	1.7542
SE-DMTG	774	0.4252	0.4397	0.2894	0.1183	0.3307	1.6033
MPC-C	2560	0.6068	0.5054	0.2793	0.2013	0.3668	1.9597
IMA-unweighted	3316	0.5822	0.5041	0.2902	0.2427	0.3838	2.0029
IMA	2561	**0.6721**	0.5342	**0.3023**	**0.2547**	**0.4232**	**2.1866**

The bold values are the highest value of each metric of each PPI network.

First, we compared them with the standard protein complexes 1. As shown in Table 4, IMA outperformed the other algorithms on the Krogan dataset. IMA obtained a F-measure of 0.6272, CR of 0.3917, MMR of 0.3266, Jaccard of 0.4373, and total score of 2.0688, which were obviously superior to other detection algorithms. MCL predicted 370 protein complexes and achieved the highest ACC of 0.3192. Second, we compared the 13 approaches using the DIP dataset. IMA detected 1338 protein complexes, and achieved the highest F-measure, MMR, Jaccard, and Total scores, respectively. ClusterONE found 904 protein complexes and achieved a CR of 0.5062 and ACC of 0.3270, the best performance in terms of CR and ACC. However, it only achieved a F-measure of 0.5118, MMR of 0.1467, Jaccard of 0.3297, and total score of 1.8214, which were lower than those obtained using the IMA method. Second, we compared our IMA and other methods using the combined6 dataset. Table 4 shows than the results obtained by using combined6 dataset are similar to those obtained using the DIP dataset. IMA detected 1054 protein complexes and achieved the highest F-measure, MMR, Jaccard, and total score with values of 0.7256, 0.3364, 0.4869, and 2.3052, respectively. IPCA found 2160 protein complexes and achieved a better CR of 0.5106. ClusterONE predicted 648 protein complexes, achieving a ACC of 0.3306, which was the highest. Finally, we also used the WI-PHI dataset to evaluate the performance of all methods, and IMA identified 2561 protein complexes, and IMA scores of F-measure, CR, MMR, Jaccard, and total score were higher than those determined by the other methods, and they were 0.7503, 0.6223, 0.3965, 0.4828, and 2.5579, respectively. As for the ACC, which is among the top three, only lower than SE-DMTG and ClusterONE. From the above analysis, we found that the IMA algorithm achieved the best performance in the most evaluation metrics, with the exception of CR and ACC in some cases. Therefore, these results demonstrate that the IMA outperforms the base and could be an excellent approach to detect protein complexes in PPI networks. More evaluation metrics are made available in the https://github.com/RongquanWang/IMA/Additional file 2.

The comparison results obtained using standard protein complexes 2 in Table 5 are basically consistent with those obtained using standard protein complexes 1 in Table 4. This means that the performance of the proposed IMA is relatively stable. The IMA algorithm performs significantly well on four PPI networks, and it is competitive with the other algorithms in term of computational evaluation metrics. Additionally, in order to further verify the performance of our IMA algorithm, we also use CYC2008 protein complex dataset and MIPS protein complex dataset to evaluate these identification algorithms. The evaluation results are shown in https://github.com/RongquanWang/IMA/Additional file 4. From the experimental results, we can see that the performance of IMA algorithm on CYC2008 protein complex dataset and MIPS protein complex dataset is basically consistent with the performance on two datasets (standard protein complexes 1 and standard protein complexes 2). This experimental results show that IMA algorithm has strong adaptability and stability to different standard protein complexes.

### 3.3 Comparison With Functional Enrichment Analysis

We needed to conduct a multi-angle analysis for this statistic, because the *p*-value of the identified protein complexes is closely related to the size of the identified protein complexes (Wang et al., 2019b). For this purpose, the number of detected protein complexes (Num), the number of significantly identified protein complexes, and the percentage of significantly identified protein complexes with different *p*-values from 1E-2 to 1E-20 were used to analyze their functional enrichment. We used a *p*-value test to analyze the protein complexes discovered by the IMA, MCODE, MCL, IPCA, COACH, ClusterONE, PEWCC, ProRank+, WPNCA, WEC, EWCA, SE-DMTG, and MPC-C. The results of the *p*-values of these methods are shown in Table 6.

**TABLE 6:** Functional enrichment of the protein complexes identified using different algorithms.

Algorithms	Num	< E-20	< E-15	< E-10	< E-5	Significant
Krogan						
MCODE	39	6 (15.38%)	8 (20.51%)	14 (35.89%)	24 (61.53%)	33 (84.61%)
MCL	370	43 (11.62%)	72 (19.46%)	125 (33.78%)	246 (66.48%)	275 (74.32%)
IPCA	582	108 (18.56%)	162 (27.84%)	244 (41.93%)	445 (76.47%)	485 (83.34%)
COACH	345	66 (19.13%)	107 (31.01%)	165 (47.82%)	272 (78.83%)	295 (85.5%)
ClusterONE	240	62 (25.83%)	92 (38.33%)	130 (54.16%)	199 (82.91%)	212 (88.33%)
PEWCC	383	144 (37.6%)	196 (51.18%)	275 (71.81%)	357 (93.22%)	374 (97.66%)
ProRank+	357	88 (24.65%)	119 (33.33%)	184 (51.54%)	283 (79.27%)	311 (87.11%)
WPNCA	369	62 (16.8%)	100 (27.1%)	167 (45.26%)	290 (78.59%)	311 (84.28%)
WEC	516	133 (25.78%)	186 (36.05%)	262 (50.78%)	421 (81.59%)	447 (86.63%)
EWCA	676	149 (22.04%)	216 (31.95%)	323 (47.78%)	529 (78.25%)	564 (83.43%)
SE-DMTG	372	80 (21.51%)	110 (29.57%)	161 (43.28%)	282 (75.81%)	301 (80.92%)
MPC-C	458	130 (28.38%)	199 (43.45%)	293 (63.97%)	442 (96.5%)	**449(98.03%)**
IMA-unweighted	767	219 (28.55%)	301 (39.24%)	457 (59.58%)	681 (88.78%)	712 (92.82%)
IMA	773	226 (29.24%)	319 (41.27%)	501 (64.81%)	719 (93.01%)	735 (95.08%)
DIP						
MCODE	26	8 (30.77%)	12 (46.15%)	14 (53.84%)	19 (73.07%)	19 (73.07%)
MCL	628	118 (18.79%)	184 (29.3%)	279 (44.43%)	443 (70.54%)	485 (77.23%)
IPCA	1242	147 (11.84%)	315 (25.37%)	556 (44.77%)	972 (78.26%)	1039 (83.65%)
COACH	329	75 (22.8%)	122 (37.09%)	177 (53.81%)	290 (88.16%)	305 (92.72%)
ClusterONE	904	137 (15.15%)	201 (22.23%)	337 (37.27%)	690 (76.32%)	772 (85.39%)
PEWCC	648	153 (23.61%)	247 (38.12%)	376 (58.03%)	572 (88.28%)	597 (92.14%)
ProRank+	167	23 (13.77%)	38 (22.75%)	63 (37.72%)	129 (77.24%)	138 (82.63%)
WPNCA	623	156 (25.04%)	242 (38.84%)	370 (59.39%)	562 (90.21%)	590 (94.7%)
WEC	253	97 (38.34%)	121 (47.83%)	149 (58.9%)	195 (77.08%)	209 (82.61%)
EWCA	964	172 (17.84%)	284 (29.46%)	477 (49.48%)	823 (85.37%)	866 (89.83%)
SE-DMTG	869	142 (16.34%)	213 (24.51%)	358 (41.2%)	708 (81.48%)	770 (88.61%)
MPC-C	1477	323 (21.87%)	538 (36.43%)	906 (61.35%)	1398 (94.66%)	1445 (97.84%)
IMA-unweighted	1569	327 (20.84%)	495 (31.55%)	810 (51.63%)	1430 (91.15%)	1492 (95.1%)
IMA	1338	382 (28.55%)	577 (43.12%)	897 (67.04%)	1305 (97.53%)	**1324 (98.95%)**
combined6						
MCODE	63	26 (41.27%)	31 (49.21%)	42 (66.67%)	57 (90.48%)	60 (95.24%)
MCL	508	129 (25.39%)	162 (31.89%)	209 (41.14%)	323 (63.58%)	349 (68.7%)
IPCA	2160	579 (26.81%)	784 (36.3%)	1145 (53.01%)	1923 (89.03%)	2027 (93.84%)
COACH	682	156 (22.87%)	196 (28.74%)	290 (42.52%)	520 (76.24%)	575 (84.3%)
ClusterONE	648	148 (22.84%)	208 (32.1%)	258 (39.82%)	420 (64.82%)	461 (71.15%)
PEWCC	737	285 (38.67%)	375 (50.88%)	505 (68.52%)	688 (93.35%)	707 (95.93%)
ProRank+	472	255 (54.03%)	324 (68.65%)	395 (83.69%)	443 (93.86%)	452 (95.77%)
WPNCA	898	375 (41.76%)	493 (54.9%)	609 (67.82%)	797 (88.76%)	829 (92.32%)
WEC	544	235 (43.2%)	273 (50.19%)	310 (56.99%)	400 (73.53%)	423 (77.76%)
EWCA	935	274 (29.3%)	337 (36.04%)	437 (46.74%)	721 (77.11%)	770 (82.35%)
SE-DMTG	490	147 (30.0%)	199 (40.61%)	248 (50.61%)	431 (87.96%)	455 (92.86%)
MPC-C	1008	311 (30.85%)	437 (43.35%)	651 (64.58%)	969 (96.13%)	993 (98.51%)
IMA-unweighted	1183	370 (31.28%)	547 (46.24%)	798 (67.46%)	1117 (94.43%)	1152 (97.39%)
IMA	1054	387 (36.72%)	557 (52.85%)	771 (73.15%)	1032 (97.91%)	**1042 (98.86%)**
WI-PHI						
MCODE	124	24 (19.35%)	29 (23.38%)	40 (32.25%)	58 (46.77%)	64 (51.61%)
MCL	772	25 (3.24%)	35 (4.54%)	74 (9.59%)	234 (30.32%)	287 (37.19%)
IPCA	2181	411 (18.84%)	550 (25.21%)	807 (36.99%)	1259 (57.71%)	1345 (61.65%)
COACH	1353	303 (22.39%)	422 (31.19%)	591 (43.68%)	921 (68.07%)	989 (73.1%)
ClusterONE	1313	198 (15.08%)	256 (19.5%)	342 (26.05%)	555 (42.27%)	635 (48.36%)
PEWCC	1813	435 (23.99%)	627 (34.58%)	906 (49.97%)	1297 (71.54%)	1363 (75.18%)
ProRank+	255	53 (20.78%)	60 (23.53%)	83 (32.55%)	145 (56.86%)	156 (61.17%)
WPNCA	1813	429 (23.66%)	594 (32.76%)	834 (46.0%)	1253 (69.11%)	1336 (73.69%)
WEC	729	215 (29.49%)	264 (36.21%)	337 (46.22%)	478 (65.56%)	501 (68.72%)
EWCA	2347	474 (20.2%)	675 (28.76%)	950 (40.48%)	1428 (60.85%)	1532 (65.28%)
SE-DMTG	774	87 (11.24%)	146 (18.86%)	255 (32.94%)	496 (64.08%)	540 (69.76%)
MPC-C	2560	452 (17.66%)	766 (29.93%)	1382 (53.99%)	2163 (84.5%)	2239 (87.47%)
IMA-unweighted	3316	715 (21.56%)	1062 (32.02%)	1656 (49.93%)	2646 (79.79%)	2815 (84.89%)
IMA	2561	847 (33.07%)	1243 (48.53%)	1732 (67.62%)	2326 (90.81%)	**2379 (92.88%)**

The bold values are the highest value of each metric of each PPI network.

As shown in Table 6, the number of protein complexes that could be significantly detected by IMA was higher than that determined by the other methods in the four PPI networks. This means that IMA can detect more protein complexes with biological significance compared to other methods. Although some detected protein complexes do not match standard protein complexes currently, they are likely to be real protein complexes. As for the percentage of significantly detected protein complexes at different thresholds of the *p*-value from E-2 to E-20 in Table 6, we can conclude that IMA could detect a relatively higher proportion of protein complexes with biologically significance in most PPI networks. The above analysis demonstrates that the IMA method could be a promising method for discovering new protein complexes with biological significance.

### 3.4 Comparison With Subcellular Location Score

According to the definition of colocalization score, it is based on the average colocalization of all detected protein complexes. It should be noted that the lower the number of detected protein complexes, the higher the colocalization score. Here, we used the ProCope tool (Krumsiek et al., 2008) to calculate the colocalization score.

Table 7 shows the average co-localization scores of protein complexes detected using various methods on localization dataset, (Huh et al., 2003). In Krogan, the best co-localization score of 0.7442 is obtained by the MCODE method, but MCODE only detected 39 predicted protein complexes, which was beneficial for achieving high the co-localization score, and IMA obtained a score of 0.7351, lower than MCODE. In DIP, ProRank + detected 357 protein complexes and obtained a co-localization score of 0.6933, which was better than that of all the other methods. In combined6, IMA method detected 1054 detected protein complexes and achieved the highest the co-localization score of 0.7440. In WI-PHI, IMA achieved the highest co-localization score, and the number of protein complexes was 2561. Based on the co-localization score of the detected protein complexes by IMA, it indicates that the proteins of protein complexes predicted by IMA have better localization consistency; these proteins in the same protein complex tend to carry out a similar function.

**TABLE 7:** The co-localization scores of protein complexes detected by different methods in four PPI networks.

Algorithms	Num	Krogan	Num	DIP	Num	combined6	Num	WI-PHI
Co-localization score								
MCODE	39	**0.7442**	26	0.6156	63	0.6481	124	0.5586
MCL	370	0.5820	628	0.5598	508	0.5182	772	0.5510
IPCA	582	0.6656	1242	0.6113	2160	0.5883	2181	0.5585
COACH	345	0.6587	329	0.6527	682	0.5833	1353	0.4960
ClusterONE	240	0.6716	904	0.5778	648	0.5277	1313	0.5207
PEWCC	383	0.7107	648	0.6271	737	0.6493	1813	0.5110
ProRank+	357	0.6779	167	**0.6933**	472	0.7377	255	0.5570
WPNCA	369	0.6245	623	0.6063	898	0.5270	1813	0.4997
WEC	516	0.7182	253	0.6520	544	0.6803	729	0.5039
EWCA	676	0.6960	964	0.6430	935	0.6707	964	0.5303
SE-DMTG	372	0.7164	869	0.6725	490	0.7247	774	0.6419
MPC-C	458	0.7315	1477	0.6503	1008	0.7178	2560	0.6111
IMA-unweighted	767	0.7052	1569	0.6556	1183	0.7090	3316	0.6199
IMA	773	0.7351	1338	0.6728	1054	**0.7440**	2561	**0.6506**

The bold values are the highest value of each metric of each PPI network.

## 4 Case Study and Discussion

**FIGURE 4:**
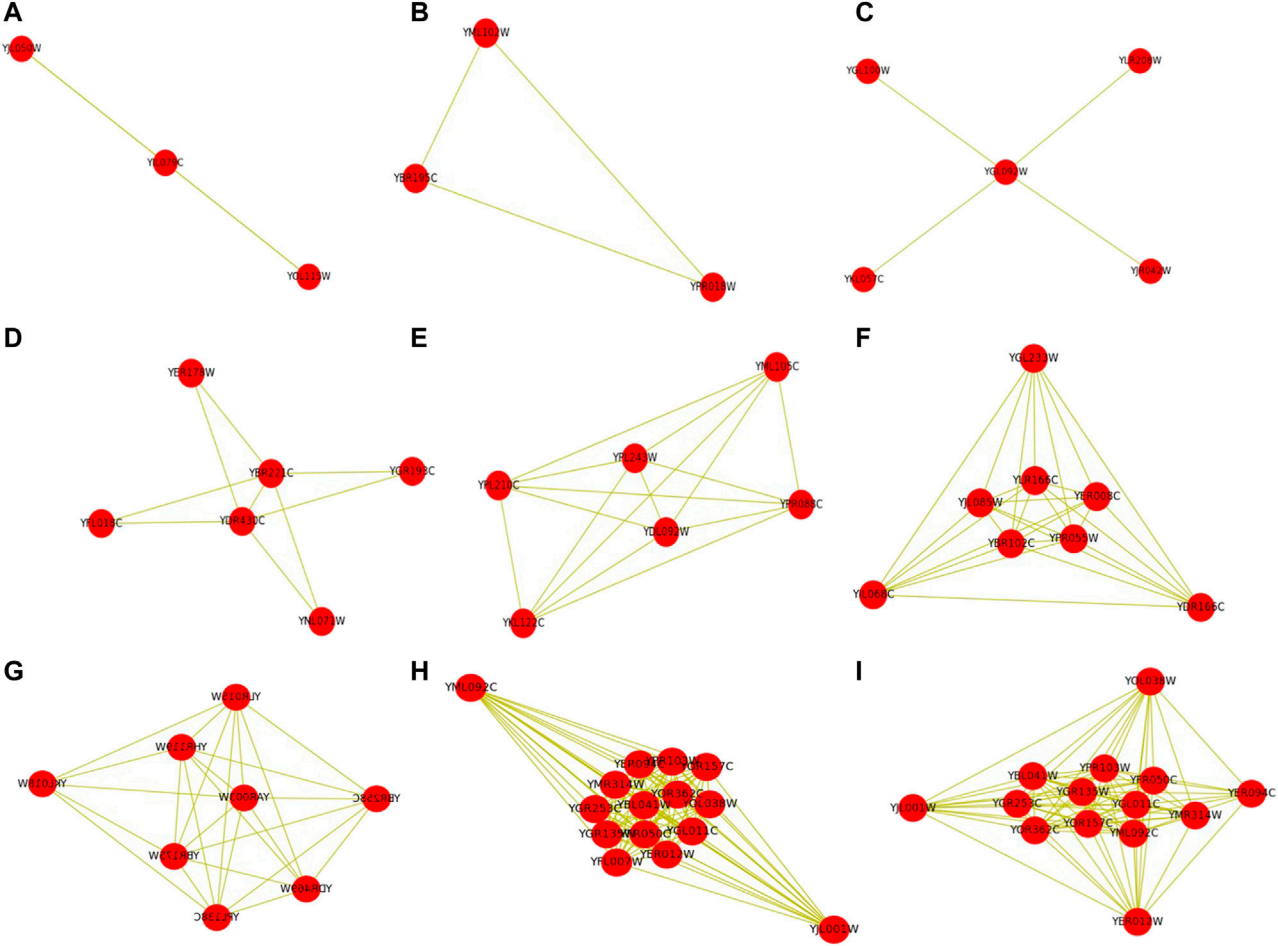
Some examples of detected protein complexes with different topological structures by IMA. Note that these protein complexes contain the number of proteins between 3 and 14. Proteins that have binary interactions are connected by dashed lines.

IMA algorithm can detect protein complexes with multiple topological structures. Figure 4 shows some examples of the detected protein complexes with different topological structures by using the IMA algorithm. Note that the standard protein complexes 1 and 2 are also detected by the IMA algorithm. These protein complexes contain the number of proteins between 3 and 14. These protein complexes with different topological structures include linear, triangular, star-like, rectangular, k-clique, dense subgraph, and core-attachment structure, and hybrid structure. Proteins that have binary interactions are connected by dashed lines. More examples can be found them at https://github.com/RongquanWang/IMA/Examples.

**FIGURE 5:**
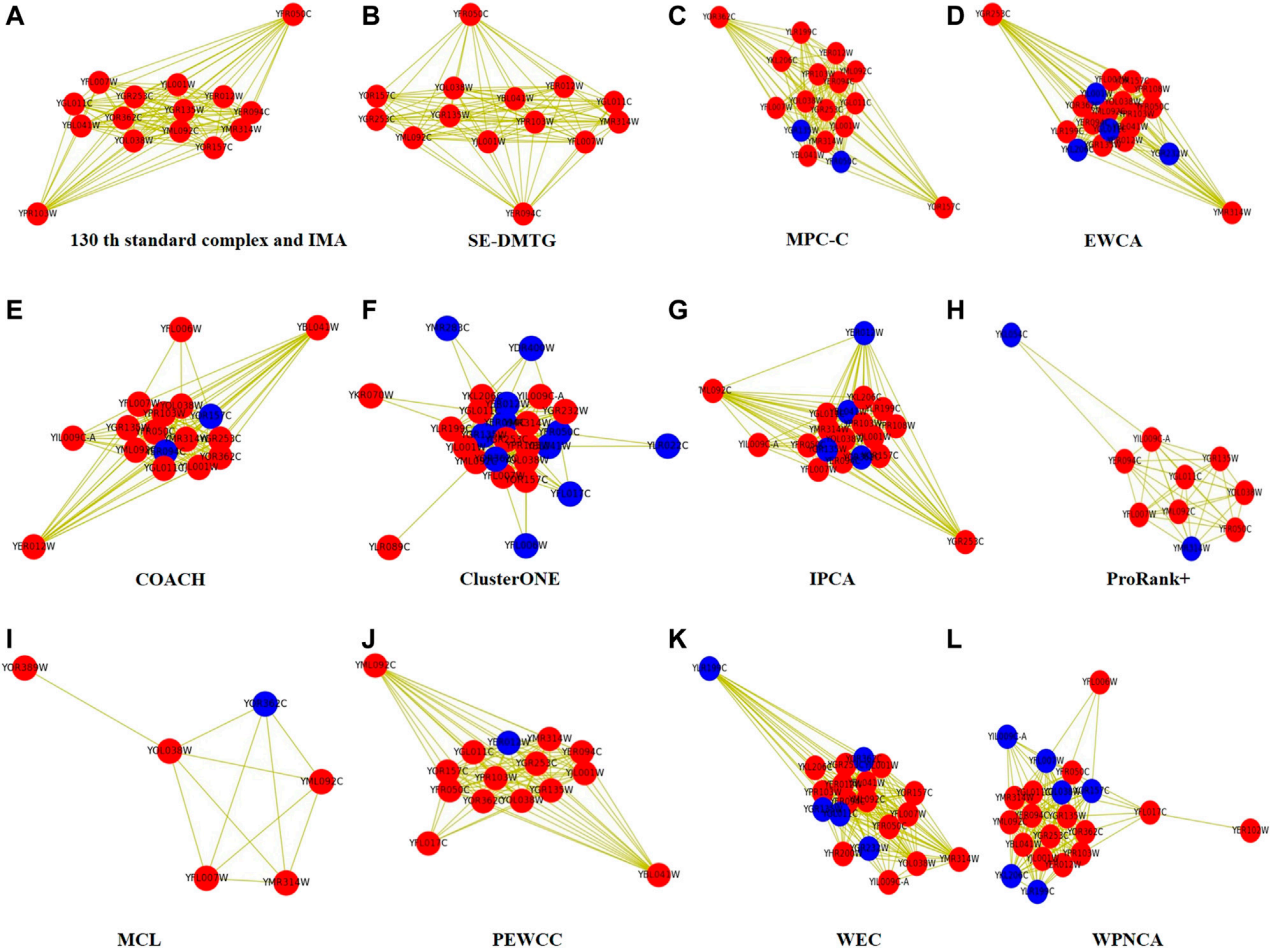
The 130^*th*^ protein complex in standard protein complexes 2 detected by different methods based on WI-PHI PPI network. Note that the red nodes represent the correctly identified proteins, and the blue nodes represent the wrongly identified proteins by method.

Figure 5 visualizes an example of the 130^*th*^ protein complex in standard protein complexes 1 in the WI-PHI dataset so as to display the detection result more clearly. Figure 5A shows that our IMA successfully detected all proteins correctly. Figures 5B–L illustrate the protein complexes identified by IMA, SE-DMTG, MPC-C, EWCA, COACH, ClusterONE, IPCA, ProRank+, MCL, PEWCC, WEC, and WPNCA, respectively. The red nodes represent the correctly identified proteins, and the blue nodes represent the wrongly identified proteins.

From Figure 5, we can see that SE-DMTG correctly identifies 14 proteins, but misidentifies a protein. Moreover, the other methods have inaccurately proteins. Our IMA can correctly identify almost all proteins, which suggests that the IMA algorithm is superior to the other comparative methods.

## 5 Conclusion

In this paper, we present a novel IMA method for identifying protein complexes in PPI network. The key idea of IMA is enabled us to design an improved memetic algorithm to optimize a fitness function for identifying protein complexes in PPI networks based on existing contending methods and a weighted PPI network. Here, an improved memetic algorithm is the cooperation of a genetic algorithm with a local optimization strategy. A genetic algorithm is used to improve the diversity of the population, and the local optimization strategy helps to locate better solutions more quickly. Furthermore, we designed a fitness function to overcome the limitations of a single objective function in estimating an individual’s fitness. The experimental results show that IMA significantly outperforms the existing outstanding algorithms in various metrics. We will use graph neural networks (Zhang et al., 2021) and other evolutionary algorithms to improve the accuracy of protein complexes identified in the future.

## Data Availability

Publicly available datasets were analyzed in this study. This data can be found here: https://github.com/RongquanWang/IMA.
